# Effects of Environmental Factors on Nutrients Release at Sediment-Water Interface and Assessment of Trophic Status for a Typical Shallow Lake, Northwest China

**DOI:** 10.1155/2013/716342

**Published:** 2013-08-05

**Authors:** Dekun Hou, Jiang He, Changwei Lü, Ying Sun, Fujin Zhang, Khureldavaa Otgonbayar

**Affiliations:** ^1^College of Life Science of Inner Mongolia University, University West Road No. 235, Saihan District, Hohhot, Inner Mongolia 010021, China; ^2^College of Environment and Resource, Inner Mongolia University, University West Road No. 235, Saihan District, Hohhot, Inner Mongolia 010021, China; ^3^Institute of Environmental Ecology, Inner Mongolia University, University West Road No. 235, Saihan District, Hohhot, Inner Mongolia 010021, China; ^4^Institute of Environmental Resources and Analytical Technique, Inner Mongolia Academy of Agricultural and Animal Husbandry Sciences, Hohhot 010031, China

## Abstract

Surface sediment and water samples were collected from Daihai Lake to study the biogeochemical characteristics of nitrogen and phosphorus, to estimate the loads of these nutrients, and to assess their effects on water quality. The contents and spatial distributions of total phosphorus (TP), total nitrogen (TN), and different nitrogen forms in sediments were analyzed. The results showed that concentrations of TN and TP in surface sediments ranged from 0.27 to 1.78 g/kg and from 558.31 to 891.29 mg/kg, respectively. Ratios of C : N ranged between 8.2 and 12.1, which indicated that nitrogen accumulated came mainly from terrestrial source. Ratios of N : P in all sampling sites were below 10, which indicated that N was the limiting nutrient for algal growth in this lake. Effects of environment factors on the release of nitrogen and phosphorus in lake sediments were also determined; high pH values could encourage the release of nitrogen and phosphorus. Modified Carlson's trophic state index (TSI_M_) and comprehensive trophic state index (TSI_C_) were applied to ascertain the trophic classification of the studied lake, and the values of TSI_M_ and TSI_C_ ranged from 53.72 to 70.61 and from 47.73 to 53.67, respectively, which indicated that the Daihai Lake was in the stage of hypereutropher.

## 1. Introduction

Along with the rapid economic development and population growth, eutrophication caused by excessive inputs of phosphorus (P) and nitrogen (N) has become one of the most common impairments of surface waters in China and presents an ongoing threat to the vitality of freshwater ecosystems, where it often manifests as harmful algal blooms that prevent sunlight from reaching underwater plants and lead to lower oxygen concentrations [1–4]. In general, nutrients are introduced into lakes and reservoirs from external sources, such as sewage discharges, agricultural wastewater, and diffuse runoff from agricultural land [5]. Over time, these nutrients could also build up in the sediment and create the potential for an internal load that can be recycled back into the water column under different environmental conditions [6]. There is also an internal source of nutrients, and it may be important as dissolved oxygen can be very low in summer [7], which will help release nutrient from sediment to water column and promote algal growth. But, the internal sources are undetermined. Such complex exchange processes of nutrients across the sediment-water interface are of considerable importance in understanding the impacts of the chemical composition and the trophic level of aquatic systems, particularly in shallow lakes and coastal marine environments [8–11]. As one of major nutrients for aquatic ecology, P is the least abundant among the nutrient needed in large quantity by photosynthetic organisms, so it is the primary nutrient that limits their growth [12]. P can also limit or colimit algal growth in estuarine and lake environments that are sustaining high N inputs [2, 13]. The phosphorus concentration in the overlying water of lake and estuarine environments, which comes from sediment, is regarded as a major component of the internal source. Such release from bottom sediment may have a significant impact on water quality and may result in continued eutrophication.

Sediments are an important source of nutrient to freshwater ecosystems [7]. Processes leading to phosphorus released to the water column from underlying sediments are numerous and include the desorption and dissolution of P bound in precipitates and inorganic materials, microbial mineralization of organic matter, and the diffusion of dissolved P form sediment pore waters [14, 15]. The environmental variables that appear to regulate the release rate of dissolved P from sediments are temperature, dissolved oxygen concentration, pH value, and redox potential [16–18]. The static releases of phosphorus from lake sediments have been examined by most experimental studies during past decades [19–22]. Because of the incompleteness of dynamic release of research, contaminated sediment released under hydrodynamic condition is a growing area of focus. Laboratory experiments such as oscillating grid, annular tank, and open water channel have been conducted to study the contaminated sediment release regularity under conditions of flowing water [23].

Classification of lakes based on various method and indices have been made by various works. Due to the importance, complexity, and variability of eutrophicated systems, mathematical models are essential tools to represent the degree of eutrophication of natural water bodies [23, 24]. However, due to various geographic sites, environment, and human activities, the assessment methods for the types of lakes eutrophication are different. The Carlson trophic status index, the modified Carlson trophic state index, nutritional index method, integrated nutrition state index method, scoring method, and other methods nowadays are presented [25–28]. Among them, the classical and most commonly used method that is based on the productivity of the water body is the biomass related trophic state index developed by Carlson [29]. Carlson's trophic state index (TSI) is a common method for characterizing a lake's trophic state or overall health. The estimation of TSI requires six physical, chemical, and biological parameters including total phosphorus (TP), total nitrogen (TN), chemical oxygen demand (COD), Secchi disk depth (SD), chlorophyll-a (Chl-a) concentration and phytoplankton biomass (CA) to know the eutrophication state of the lake environment. The average values of TSI of these six parameters will be considered in determining Carlson's trophic state index. The TSI offers a 0–100 scale providing continuous numerical classes of lake trophic states and a rigorous foundation for quantitative studies of mechanisms behind the eutriophication.

The Lake Daihai is a typical algal-dominated lake, and there are about 121 species and 76 genera of algae of which belong to Chlorophyta (28 genera), Bacillariophyta (21 genera), Cyanophyta (9 genera), Euglenophyta (7 genera), and other algae including Pyrrophyta, Cryptophyta, and Chrysophyta [30]. In addition, there is a small area of *Phragmites communis Trin* growing in the northwest and south shores, and eutrophication has appeared in Lake Daihai, which have had a negative impact on economic and social development. Consequently, the degree of eutrophication should be determined, and the causation of eutrophication should be diagnosed in the process of eutrophication management, which will be helpful to achieve effective plan and management. The purpose of this study was to analyze the spatial distribution of nitrogen and phosphorus in surface sediment and estimate sediment nutrient release properties. Additionally, two types of eutrophication evaluation methods were applied to analyze the trophic state of Daihai Lake. This study will provide helpful information for the practices of endogenous release estimation and for the improvement of engineering approach to reduce the release intensity.

## 2. Materials and Methods

### 2.1. Study Area

**Figure 1:**
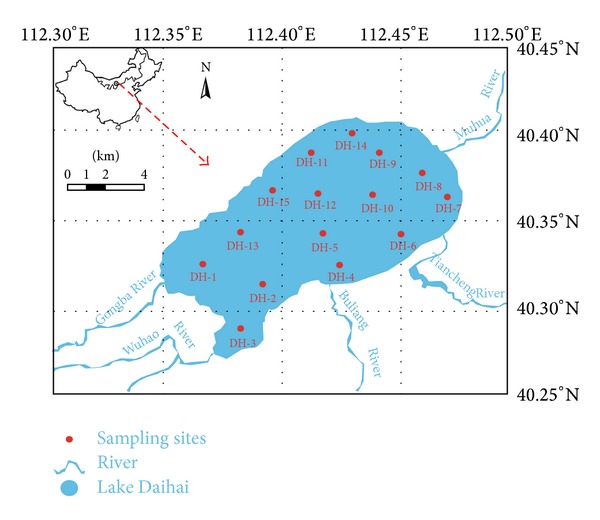
Map of the Daihai Lake with sampling sites.

The study was conducted in the Inner Mongolia Plateau of Lake Daihai (112°33′31′′~112°46′40′′E; 40°29′27′′~40°37′06′′N, 1220 m asl), located at 10 km east of Liangcheng County, northwest China (Figure 1). This ellipsoid lake has a maximum length of 20 km, a maximum width of 14 km, and water surface area of ca. 160 km^2^. The maximum and mean water depth is of 16.1 m and 7.4 m, respectively [31]. The alkalinity of the lake water is 10.8 mmol/L, and pH typically ranges between 8.79 and 9.01. The climate of the Lake Daihai basin is affected in winter by cold, continental high-pressure systems, while in summer, warmer and moister air, originating from ocean evaporation at lower latitudes, reaches the lake area and brings more precipitation [32]. Mean annual precipitation is about 350–450 mm, occurring mainly during the rainy season from July to September, and accounts for ca.66% of the mean annual precipitation. The mean annual evaporation is of 1938 mm, primarily concentrated from April to July. The vegetation assemblage changes from arid forest to shrub grassland, to semiarid grassland. The source of intake for the lake is mainly from precipitation and intermittent rivers around the lake. The amount of industrial wastewater from small industrial enterprises in the basin flowing into the lake through the Gongba River is very little; therefore, the agricultural nonpoint source pollution is the main pollution source of the lake [33].

### 2.2. Sampling and Analysis Methods

Location of sampling sites in the Lake Daihai was set according to the book entitled *Lake Ecosystem Observation Method*, and the sampling sites were given in Figure 1. The samplings in this area were carried out in September of 2007. Water samples for nutrient determination were collected at a depth of 0.5 m beneath the water surface in plastic bottles pretreated overnight with 1 M HCl solution and rinsed afterwards twice with redistilled water. At each site, two parallel water samples were collected, one of which. After filtration through 0.45 *μ*m nitrocellulose membrane, filters were stored at −20°C until the time of analysis. Surface sediments samples were taken by a grab sampler at a depth of 0–10 cm which was quickly packed in air tight polythene bags and transported to the laboratory for storage at −20°C until the analytical determinations. During the sample collection, a global positioning system (GPS) was used to locate the sites. In addition, the water quality monitoring at the studied lake was also carried out, pH, salinity, temperature, dissolved oxygen, and the Secchi depth in situ.

Sediment samples were oven-dried at 60°C to constant mass for approximately 48 h, and then the coarse debris and gravel were removed by passing the dried sample through a 2 mm sieve. The sieved samples were ground to powder with a mortar and pestle, then sieved using a 63 *μ*m mesh, and homogenized prior to laboratory analyses. Sediment total nitrogen (TN) was determined by the Kjeldahl nitrogen method. Total phosphorus (TP) was determined by spectrophotometer method after digesting with HNO_3_-HCl (3 : 1, V/V) at 200°C. Triplicates of every sample were analyzed, and the concentrations of TN and TP used here were the average of two measurements. The overall analytical precision was determined at ±5% for TN and ±3% for TP. Inorganic nitrogen (IN) in lake sediment is primary NH_4_^+^-N and NO_3_^−^-N, and the content of NO_2_^−^-N is small and can be left out of TN [34]. To determine the contents of NH_4_^+^-N and NO_3_^−^-N in lake sediments, 0.5 g dried sediment samples were added into 100 mL acid-washed screw-cap polyethylene centrifuge tubes with 2 mol/L KCl solution. The tubes were capped and incubated at 25 ± 1°C in an orbital shaker at 200 rpm for 2 h. After homogenization, the sample solution was immediately centrifuged at 5000 g for 15 min and then filtered through 0.45 *μ*m GF/C filter membrane. The filtrate was extracted for NH_4_^+^-N and NO_3_^−^-N analyses.

 Water samples from the Lake Daihai were analyzed by the following procedures. The unfiltered waters were used to determine the concentrations of TP and TN. Total nitrogen was measured by converting all nitrogen forms to nitrate by alkaline persulfate oxidation and subsequent analysis of nitrate by 2,6-dimethylphenol method. The phosphorus concentrations of all samples were analyzed spectrophotometrically by ammonium molybdate method of Murphy and Riley [35] using ascorbic acid as a reducing agent. The filtered waters were analyzed for the concentrations of nitrite-N, nitrate-N, ammonia-N, and ortho-P. Ammonia-N was determined by indophenol-blue method, while nitrite-N and nitrate-N by cadmium reduction. Chlorophyll-a in water sample was analyzed with spectrophotometry.

### 2.3. Nutrients Release Experiments

#### 2.3.1. Effects of Environmental Factors on Phosphorus Release at the Sediment and Water Interface

Phosphorus release experiments were performed according to the book entitled *Standard methods in Lake Eutrophication investigation* [36]. Ten grams of wet sediment sample was put into a 500 mL Pyrex beaker containing 250 mL overlying water form Daihai Lake, the height of the water column was recorded, and the height ratio of sediment and water was about 1 : 10. In order to simulate various conditions for comparing nutrient release to assess the impact of the key environmental boundary conditions such as light, pH, dissolved oxygen, temperature, and other parameters that are of relevance to the release experiments, effects of light and temperature on phosphorus release was controlled using an illumination incubator, and the dark/light cycle was 12 : 12 h. In the dark condition, the temperature was set at 20°C according to the temperature of Daihai Lake. In the presence of light, the light intensity was controlled by a light source of 400–700 nm measured at the surface of the beakers. The intensity of disturbed was simulated using a stirrer at different speeds. Redox conditions (related to dissolved oxygen) were controlled by the circulation of air every day and of nitrogen for 2 h after daily sampling (aerobic conditions: *ρ*DO > 5 mg/L; anaerobic conditions: *ρ*DO < 0.8 mg/L). The pH values were adjusted using 1 mol/L HCl or 1 mol/L NaOH solution. To minimize splashing and evaporation, all the beakers were covered with plastic film. All experiments were conducted in parallel. The overlying water samples (20 mL) were collected during the experiments every day, the water samples were collected 5 cm above the sediment-water interface using a syringe, and then 20 mL filtered lake water was added to maintain the total overlying water volume constant. In order to obtain the amount of release phosphorus, the following equation was employed:

(1) $$\begin{array}{c} \gamma =\frac{\left[V\left(C_{n}-C_{0}\right)+\sum V_{n}\left(C_{n-1}-C_{\text{mix}}\right)\right]}{10}, \end{array}$$

where *γ* is the amount of phosphorus release (mg/kg); *V* is the volume of overlying water (0.25 L in this study); *C*_*n*_ is the concentration of total phosphorus got the *N*th time; *C*_0_ is the initial concentration of total phosphorus; *C*_mix_ is the concentration of total phosphorus after replenishing overlying water; *V*_*n*_ is the sampling water volume each time; *n* is the number of sampling.

#### 2.3.2. Effects of Environment Factors on Ammonium Release Kinetic Experiments

It is well known that there are many ions in the overlying water of shallow lakes, such as K^+^, Ca^2+^, Mg^2+^, and Cl^−^SO_4_^2−^. Therefore, according to the previous studies [31, 37], 0.02 M KCl solution was used as the simulative lake water with some ionic strength. In order to better study the effects of environmental factors on NH_4_^+^-N release, the experiments were conducted in transparent glass centrifuge tubes. A series of 100 mL acid-washed screw-cap tubes with 25 mL overlying water were added with 5.0 g dried samples. The tubes were capped and placed at 25 ± 1°C in an orbital shaker for a series of time intervals between 0 and 48 days at 60 rpm. The sampled solution was immediately centrifuged at 5000 rpm for 15 min and then filtered through 0.45 *μ*m GF/C filter membrane. The filtrate was extracted for NH_4_^+^-N analysis using colorimetric method. All samples were analyzed in triplicate, and the data were expressed as the average.

### 2.4. Models of Eutrophication Evaluation

#### 2.4.1. Modified Trophic State Index (TSI_M_)

Modified trophic state index (TSI_M_) which was proposed by Aizaki et al. [38] had been used widely to evaluate the degree of nutrient contamination or pollution in aquatic and marine environment, and the modified trophic state index of can be calculated by the following formulas:

(2) $$\begin{array}{c} \text{TS}{\text{I}}_{\text{M}}\left(\text{chl-a}\right)=10\left(2.46+\frac{\ln\left(\text{chl-a}\right)}{\ln2.5}\right),\\ \text{TS}{\text{I}}_{\text{M}}\left(\text{SD}\right)=10\left(2.46+\frac{3.69-1.53\ln\left(\text{SD}\right)}{\ln2.5}\right),\\ \text{TS}{\text{I}}_{\text{M}}\left(\text{TP}\right)=10\left(2.46+\frac{6.71+1.15\ln\left(\text{TP}\right)}{\ln2.5}\right), \end{array}$$

where TSI_M_ is the Carlson modified trophic state index and ln is natural logarithm. Total phosphorus and chlorophyll-a are in mg/L, and SD transparency is in meters. A range between 30–50 is usually associated with the mesotrophy (moderate productivity); index values greater than 50 are associated with eutrophy (highly productivity); values less than 30 are associated with oligotrophy (low productivity).

#### 2.4.2. Comprehensive Trophic State Index (TSI_C_)

The comprehensive trophic state index (TSI_C_) was calculated according to the following formula:

(3) $$\begin{array}{c} {\text{TSI}}_{\text{C}}=\sum_{j=1}^{m}W_{j}•{\text{TSI}}_{j},\\ W_{j}=\frac{r_{ij}^{2}}{\sum_{j=1}^{m}r_{ij}^{2}}, \end{array}$$

where *r*_*ij*_ is the correlation coefficient between chlorophyll-a and some physicochemical variables (total phosphate, total nitrogen, and Secchi disk transparency), *W*_*j*_ is weight of each evaluating parameter, *m* is the number of parameters, and TSI_*j*_ is trophic state index converted from the value of some physicochemical variables. TSI_*j*_ is calculated (shown in Table 1) according to the method of Jin et al. [39]. The degree of eutrophication is assessed in terms of five contamination classes based on the increasing numerical value of the index as follows: TSI_*j*_ < 30 Oligotropher, TSI_*j*_ 30–50 Mesotropher, TSI_*j*_ 50–60, Light eutropher, TSI_*j*_ 60–70 Middle eutropher, and TSI_*j*_ > 70 Hypereutropher.

## 3. Results and Discussions

### 3.1. Chemical Characteristics of the Water Column and Sediment

The physical and chemical characteristics of Daihai Lake water and sediments, measured during sampling events, were shown in Table 2. It was observed that the average pH of water sample varied between 8.18 and 8.84 but did not fluctuate drastically across sampling sites, indicating slightly alkaline to medium alkaline. Salinity was also consistent across sampling sites, ranging from 0.23 g/kg to 0.44 g/kg. Surface water temperature in the studied lake averaged 23.1 ± 0.84°C, and there were no significant differences among the sampling sites. Dissolved oxygen concentrations ranged from 2.81 to 4.83 mg/L, and dissolved oxygen (DO) and temperature were inversely correlated in all sampling sites. Measured DO concentration was lower in high temperature site, and the depletion of oxygen level in these sites could possibly be the influence of biological activities and reduce oxygen gas solubility supported by the warmer temperature. Moreover, when water temperature becomes high, DO holding capacity of water decreased due to rapid saturation. Electric conductivity at the Daihai Lake were found be consistently varied between 8.19 ms/cm and 9.78 ms/cm, the lowest conductivity value was measured at site of DH-10, and the highest conductivity value was found at DH-4. Chemical oxygen demand (COD) in the studied lake was between 17.12 and 20.73 mg/L. Contents of TN, TP, and Chl-a in the water column were in range of 2.52–2.87 mg/L, 0.06–0.80 mg/L, 2.60–3.48 mg/L, and 17.12–20.73 mg/L, respectively. 

Overall, Lake Daihai sediments had relatively low organic carbon content, in the range of 6.84–23.5 g/kg, probably because of high precipitation of CaCO_3_, high erosion in the drainage area, and efficient mineralization of organic matter due to the intensive mixing of the water column. All samples which contained a water content of approximately 60% decreased significantly with the depth of sediment. The particle size distributions of the selected sediment samples were dominated in the fraction of silt, accounting for 62.6–86.1% of the total. The clay fraction was the minor fraction, accounting for 3.72–9.53% of the total.

### 3.2. Horizontal Distribution of Nutrients (N, P) in Surface Sediment of the Daihai Lake

**Figure 2:**
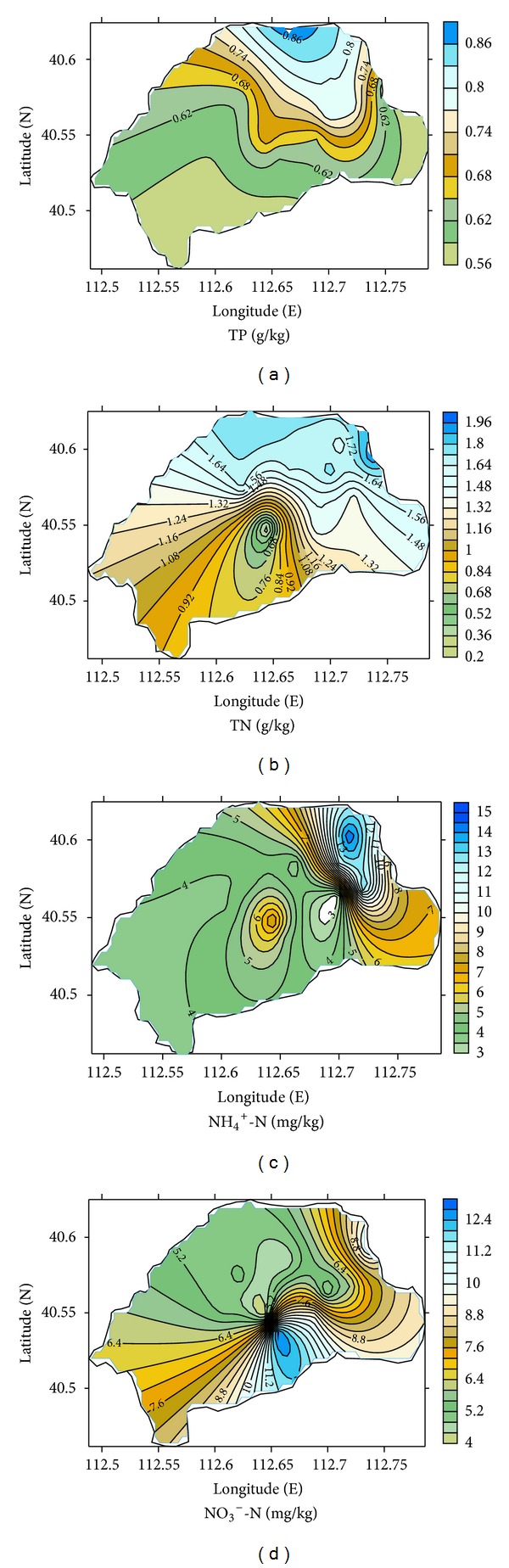
Horizontal distribution of TN, TP, NH_4_^+^-N, and NO_3_^−^-N in the surface sediments of the Lake Daihai.

Nutrient contents of sediments were controlled by numerous factors, such as the rate of sedimentation, sediment type, amount and type of organic matter, intensity of mineralization of organic matter in the sediment and water column, and redox conditions in sediment and near bottom water [40]. Results of the Lake Daihai sediment for TP, TN, NH_4_^+^-N, and NO_3_^−^-N concentrations in the surface sediment were given in Figure 2. Spatially, TP concentrations in the surface sediment of Daihai Lake were generally high, ranging from 558.31 mg/kg to 891.29 mg/kg with a mean of 708.82 mg/kg, which indicated that the lake sediments had a great potential to supply phosphorus to the overlying water. The maximum values of TP were observed in the sediment at site DH-14, which is located in a district with a high density of population and industry. Site DH-14 was also located in a scenic ecotourism area; therefore, the phosphorus content at site DH-14 was likely affected by the human activities. 

As shown in Figure 2, the contents of TN and nitrogen forms in surface sediments obviously varied. These wide ranges could be attributed to differences in the sedimentation environments. The TN concentrations in surface sediments ranged between 0.27 g/kg and 1.78 g/kg with a mean of 1.32 g/kg, and it showed a gradually decline trend from northeast to southwest of the lake. The high concentration of TN was largely due to domestic and industrial sewage discharges. Concentrations of NH_4_^+^-N fraction ranged from 2.56 to 14.5 mg/kg with a mean of 12.8 mg/kg, whereas NO_3_^−^-N ranged from 1.79 to 12.5 mg/kg. The NH_4_^+^-N and NO_3_^−^-N contributed about 51%–76% and 21%–37% of inorganic nitrogen in the sediments, respectively. That means that the primary nitrogenous component of inorganic nitrogen is NH_4_^+^-N (over 50%), and relative contribution of NH_4_^+^-N and NO_3_^−^-N to inorganic nitrogen among the studied sediment samples is stable. The inorganic nitrogen content in the sediment was controlled by its redox condition and microorganism. The intensive nitrogen transformation occurred at sediment-water interface, and the process wherein organic nitrogen was transformed into NH_4_^+^-N was ammonification, only the part of NH_4_^+^-N in sediment could be transformed into NO_3_^−^-N, when sediment was under aerobic condition [41, 42]. Therefore, the NH_4_^+^-N content in surface sediments remained higher, and its relative contribution to inorganic nitrogen is over 50%. The relative high value of NH_4_^+^-N content was found in northeast of the lake, mainly due to these sites located in deep water zone. The anaerobic conditions inhibited nitrifying activity and favored increasing NH_4_^+^-N content, and the high alkaline condition (8.2 < pH < 8.9) greatly favored ammonia volatilization in these sites. The transformation from NH_4_^+^-N to NH_3_-N was regulated by the pH of the water column [43], and large amount of transformations between NH_4_^+^-N and NH_3_-N occurred at pH values from 8 to 9 [44, 45]. In addition, higher plant litter inputs in northern of the lake favored NH_4_^+^-N accumulation in surface sediment with high clay contents, since NH_4_^+^-N was easy to be adsorbed by clay particles. Relatively high concentration of NO_3_^−^-N was found at site DH-4, and strong nitrification of NH_4_^+^-N greatly increased NO_3_^−^-N contents in this site. In addition, low water depth may reduce the possibilities for nutrient retention through plant uptake and nitrogen removal though denitrification.

**Figure 3:**
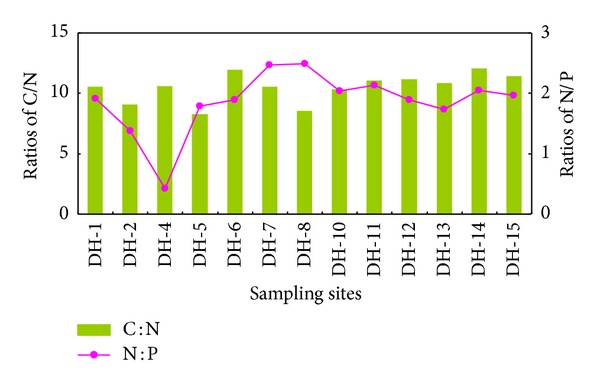
The ratios distribution of C/N and N/P in surface sediments of the Lake Daihai.

A nutrient ratio indicates whether the source of organic matter is autogenetic or allochthonous. If all the organic matters in the sediment come from phytoplanktons, the ratio of C : N : P would be close to the redfield value (106 : 16 : 1), and the ratio of C : N would be about 6.6; If they are from a terrestrial source, the ratio of C : N would generally be >20. The spatial distribution of the C : N ratio in the surface sediment of the Lake Daihai is fairly uniform with values ranging between 8.2 and 12.1 (Figure 3). It is much greater in the north part of the lake than that in south because there is more terrestrial matter intake of the sediments from rivers. In addition, more autogenic organic matters are received in these sites sediments resulting in low C : N ratio. Nitrogen is more easily degraded than carbon, thus the importance of the accumulation of organic matter, reflecting intense biological activity, with organic decomposition and nitrogen release into the water predomination. This process could be the cause of high C/N values recorded for most of the lake. 

**Figure 4:**
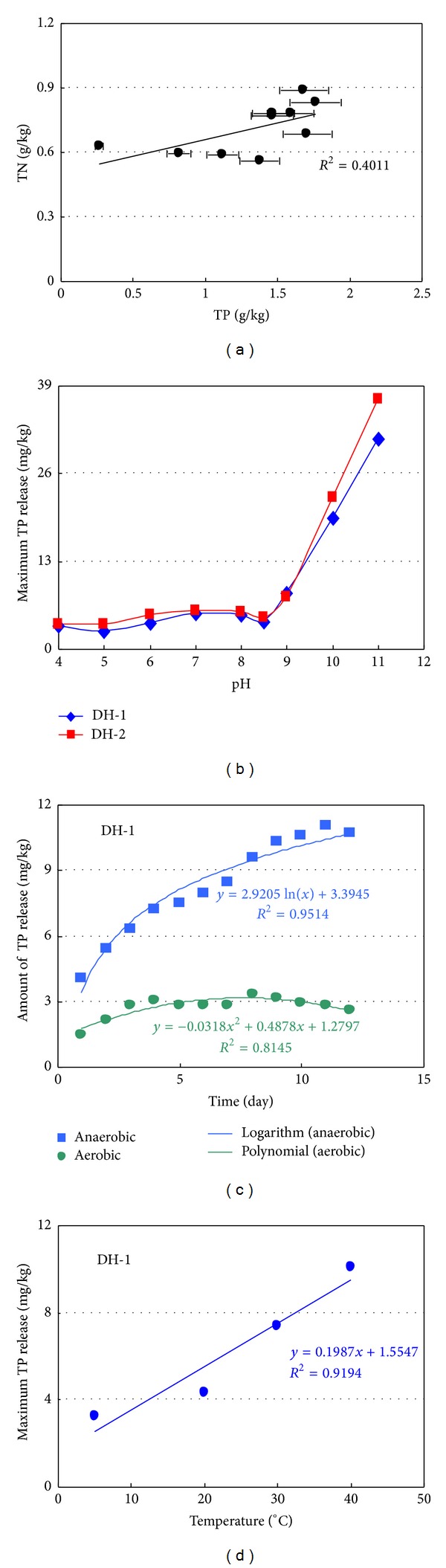
(a) The relationship between TN and TP in surface sediment of the Lake Daihai. (b) The relationship between pH and concentrations of TP released from surface sediments. (c) The release curves of TP under aerobic or anaerobic conditions. (d) The relationship between temperature and maximum concentration of TP release.

Nitrogen and phosphorus are important variables for classification of trophic state because they are the nutrient most likely to limit aquatic primary producers in rivers and lakes. Lake water total N (TN) to total P (TP) ratio is commonly used as an index that represents the nutrient limitation for algal growth. Elser et al. [46] reports that P is limiting when TN : TP by weight is >16, N is limiting when TN : TP is <14, and either N or P or both are limiting when TN : TP is between 14 and 16. This study shows that the TN : TP ratio in water column is ranged from 0.42 to 2.59 (Figure 3), suggesting that the limiting nutrient for the algal growth in the Lake Daihai is N. If nitrogen and phosphorus in the sediments are generated from the same source, they should have a good correlation. However, it is indicated that the correlation between nitrogen and phosphorus is weak in the surface sediment of the Lake Daihai, showing different sources (Figure 4(a)). The ratio of N : P in sediment is far less than the redfield value, showing that phosphorus in the studied lake sediment is mainly land derived.

Table 3 shows the Pearson correlation coefficients among C, N, P, and different ratios of nutrients in surface sediment. An excellent correlation existed between total organic carbon (TOC) and total nitrogen (TN) in the sediments of the studied lake (*r* = 0.939, *P* < 0.01) which suggested that the concentration of TN might be regulated by organic source. There was a relatively weak correlation between TOC and inorganic nitrogen (IN), which meant that the accumulation of organic nitrogen in the sediments was dominated. Because of the strong correlation between TOC and TN, it could be inferred that any increase or decrease in total organic matter will be associated with similar change in organic nitrogen, and the inorganic nitrogen contents of the samples may be presumed to be constant. 

### 3.3. Effect of Environmental Factors on Nutrients Release in Sediments

#### 3.3.1. Influencing Factors on NH_4_^+^-N Release

**Figure 5:**
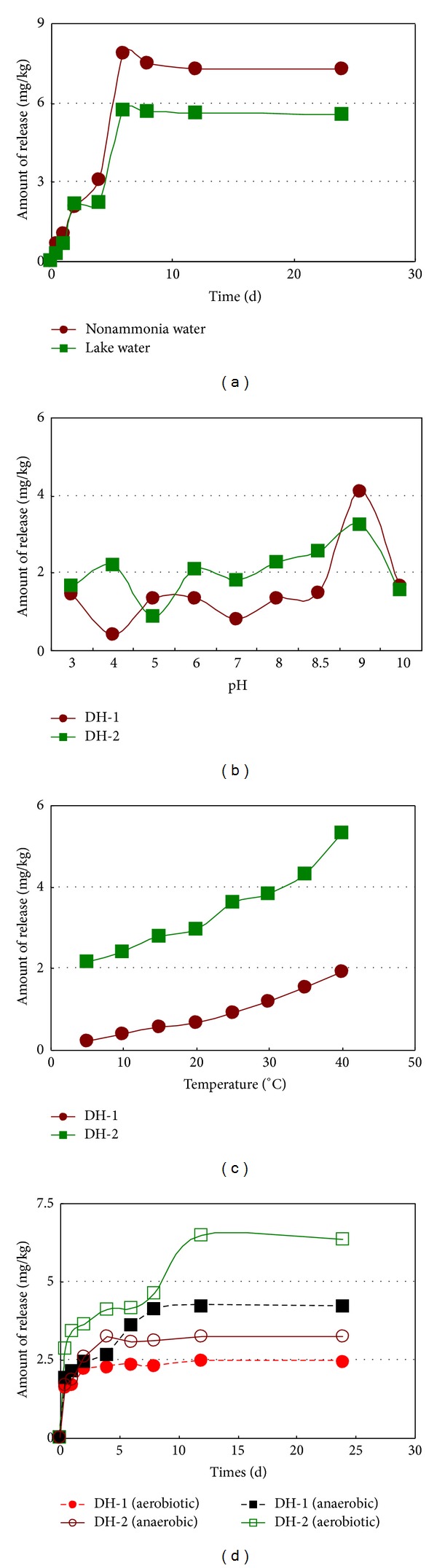
Impacts of different environment factors on NH_4_^+^-N release from surface sediments.

The process of release of nitrogen from sediments was complex, involving the interconversion of various nitrogen species. The factors which may influence the NH_4_^+^-N release kinetic curves from the sediment samples were shown in Figure 5. It could be observed that, before the process of NH_4_^+^-N release reached equilibrium, the amounts of NH_4_^+^-N released increased rapidly within the first 8 days and then gradually decreased and reached equilibrium (Figure 5(a)). The NH_4_^+^-N contributed over 60% to the inorganic nitrogen released during the experiment, which means that the NH_4_^+^-N is the main form of the inorganic nitrogen released from the sediments in the Lake Daihai. This result may relate to the mineralization of organic nitrogen into NH_4_^+^-N [47]. Relatively high pH may change the sediment properties, the affecting NH_4_^+^-N release from lake sediments. The release rate of NH_4_^+^-N from the lake sediments as a function of pH is illustrated in Figure 5(b). In general, the rate of NH_4_^+^-N release decreased with the increasing pH and remained at a low level in acidic condition, but in alkaline condition, the rate of NH_4_^+^-N release increased with the increasing pH. When pH = 9.0, the rate of NH_4_^+^-N release reached highest value (4.09 mg/kg). These observations suggest that NH_4_^+^-N release from the sediments occurred in both acidic and alkaline conditions, and alkaline condition was more favorable. Neutral condition was also in relative low level. The effect of pH on NH_4_^+^-N release was mainly reflected through the equilibrium of NH_3_H_2_O <=> NH_4_^+^ + OH^−^. The competition for sorption sites between NH_4_^+^-N and inorganic cations may be the reason why the amount of NH_4_^+^-N released dropped later. Temperature is one of the most important ecological parameters influencing the process of NH_4_^+^-N release. The amount of NH_4_^+^-N released presents an obvious increasing tend when the temperature rises. Contents of NH_4_^+^-N at 5°C are decreased by one order of magnitude compared at 40°C (e.g., 0.13 mg/kg versus 1.31 mg/kg in DH-1). Dissolved oxygen content and nitrification rate are also significantly correlated to sediment temperature. Oxygen uptake by the sediments shows a clear seasonal pattern, with maximum uptake during the summer and minimum uptake during the winter and early spring [48]. Under anaerobic conditions (*ρ*DO < 0.8 mg/L), the release of NH_4_^+^-N from surface sediment is greater than those under aerobic conditions (*ρ*DO > 5 mg/L) and reached equilibrium after 12 days of release. The nitrifying bacteria can transform great amount of NH_4_^+^-N into NO_3_^−^-N in water under high dissolved oxygen via NH_4_^+^ + 1/2O_2_ → NO_2_^−^ + 2H^+^ + H_2_O, NO_2_^−^ + H_2_O → H_2_O : NO_2_ → NO_3_^−^ + 2H^+^, and these transformations offset some of the growth on NH_4_^+^-N concentration which is released from the sediments.

#### 3.3.2. Effects of Environmental Parameters on P Release

**Figure 6:**
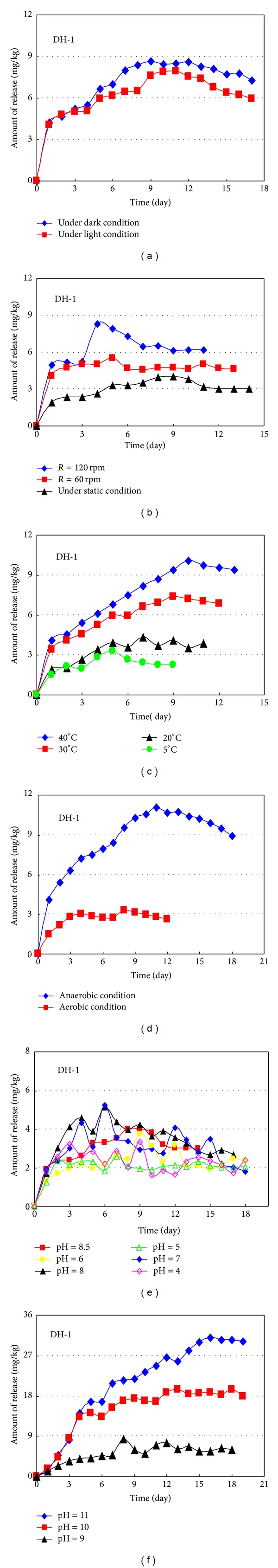
Impacts of different environment factors on phosphorus release from surface sediments.

The releases of phosphorus from sediments are influenced by various environmental parameters including redox status, temperature, disturbed, light intensity, and pH. The enhanced release of sediment phosphorus under anaerobic conditions is well documented. In this study, the overlying water did not become completely anaerobic after treatment with N_2_, because the overlying water exhibits a final dissolved oxygen concentration of 2 to 4 mg/L. The dynamic curves of P release from sediments in the aerobic and anoxic conditions were shown in Figure 6(e). The process of phosphorus release reached equilibrium in DH-1 sediments at the first 8 days in the aerobic condition and 12 days in the anoxic condition with a maximum release of 3.04 mg/kg and 11.07 mg/kg, respectively. Amount of phosphorus release can be calculated by the regression equation (Figure 4(c), aerobic: *y* = −0.0318*x* ^2^ + 0.4878*x* + 1.2797, *R* ^2^ = 0.8145; anoxic: *y* = 2.9205ln⁡(*x*) + 3.3945, *R* ^2^ = 0.9514). P can be released from the sediment to overlying water in both aerobic and anoxic conditions; however, the P release quantities were different. Relatively low dissolved oxygen concentrations in the overlying water usually increase rates of phosphorus released from sediments. The observed release was likely the result of Fe(III) reduction at the sediment-water interface. This reduction may occur even when the overlying water contains measurable oxygen concentrations if the rate of oxygen utilization at the interface is high enough to create an anoxic zone at the sediment surface [49]. The dissolved oxygen concentration at the sediment and water interface controlled the redox potential. In aerobic condition, phosphorus can bind with Fe^3+^ to form Fe_2_(PO_4_)_3_; at the same time, dissolved phosphorus in the overlying water can be adsorbed by Fe(OH)_3_ in the sediment, so it was difficult for phosphorus to release from sediment at the aerobic condition. While in the anoxic condition, phosphorus bound to Fe^3+^ could be easily released from the sediments to the overlying water. If sediments contained relatively large populations of benthic organisms, the process of phosphorus release would be altered greatly. In aerobic conditions, Fe/Al-P was strong adsorbed into the surface sediments with a high dissolved oxygen concentration, resulting in a significant limitation of mobility and release of Fe/Al-P and affected the growth of organisms by nutrient limitation. While in anoxic condition, the Fe/Al-P released stimulated the growth of organisms, which in turn stimulated the release of other phosphorus fractions. 

The concentration changes of phosphorus in the light effect experiment are shown in Figure 6(a). No significant differences for phosphorus concentrations were observed in both dark and light conditions. This indicated that the illumination and its intensity had no major effect on phosphorus release from sediment. But, it is the key factor for growth of alga in the sediment-water system [50]. The release process of phosphorus at the sediment-water interface in the dark condition was mainly influenced by bacteria, resulting in an increase in dissolved inorganic phosphorus in the overlying water. While in the light condition, this process was affected not only by bacteria but also by benthic alga and phytoplankton. So, total phosphorus in the overlying water under the dark environment was higher than that under the light condition (Figure 6(a)).

Effects of temperature on phosphorus release from the sediments are shown in Figure 6(c). The concentration of total phosphorus increased obviously with temperature increasing. The maximum release of phosphorus at 20°C, 30°C, and 40°C, respectively, was 4.34 mg/kg, 7.39 mg/kg, and 10.11 mg/kg, and the concentration of phosphorus at 5°C (3.27 mg/kg) was only a third of that at 40°C. There was a good relation between the maximum phosphorus release and temperature (Figure 4(d), *R* ^2^ = 0.9194). Temperature increases and lowed dissolved oxygen concentration in the water column also led to increased phosphorus rates. Temperature obviously affected the biomass and growth of bacteria and autotrophic alga. As temperature increased, the activity of bacteria, benthic alga, and phytoplankton was enhanced, and the increased bioturbation was beneficial to the release of phosphorus from the sediments. Moreover, dissolved oxygen was depleted with the bacteria respiration increasing in the sediment at higher temperature, which may result in the occurrence of low potential and then induce the reduction of Fe(III) from Fe(OOH) to Fe(II) and finally resulted in Fe/Al-P release. Thirdly, higher temperatures increase the mineralization rate of fresh easily degradable organic material, and a higher mineralization rate demands more oxygen which in turn lowers the amount of available oxygen thereby boosting the release of Fe/Al-P.

Disturbing intensity was one of the important factors that affected the phosphorus release from sediments (Figure 6(b)). The maximum phosphorus release (8.31 mg/kg) from DH-1 sediment under high disturbing intensity (*R* = 120 rpm) was 1.5 and 2.0 folds higher than that of under low disturbing intensity (*R* = 60 rpm) and static status, respectively. In general, phosphorus released increased with increased stirring for the Lake Daihai sediments up to point where sediment suspension occurred. Total phosphorus in the water column increased with disturbing, indicating that much of the released phosphorus was associated with particulates when sediment suspension occurred. The adsorption of phosphorus on Fe(III) hydrous oxides, formed when dissolved Fe(II) or surface Fe(II) on particulates resuspended from anoxic zones came into contact with oxygenated water, could be the explanation for the observed results.

Effects of pH on phosphorus release were shown in Figures 6(e)-6(f). There was no significant variation (2.57 mg/kg–5.26 mg/kg) on the rate of phosphorus release when pH was in range of 4.0–8.5. The concentration of total phosphorus in the overlying water remained lower or was close to the detection limit, and the minimum rate was found at pH = 5. The rate of phosphorus release tend to increase with pH increasing when pH > 9, and the rates of phosphorus release reached 31.03 mg/kg and 37.04 mg/kg at pH = 11 (Figure 4(b)), respectively. Higher pH may change the sediment properties, thus affecting the phosphorus release from the sediments. The effect of pH on phosphorus release was mainly reflected through the phosphorus speciation in combination with metals such as Fe, Al, and Ca [16]. The NaOH-P represented phosphorus bound to metal oxides (mainly Al and Fe) and was exchangeable with OH^−^ and other inorganic phosphorus compounds soluble in bases. The capacity of phosphorus binding iron and aluminum compounds decreased as pH increased in the overlying water, and in the sediment, relatively high pH promoted the release of NaOH-P. pH had also an important secondary effect on phosphorus release from the lake sediment. If iron (II) and orthophosphate were released from an anoxic sediment surface and were mixed into aerobic lake water at a high pH, only part of the released phosphorus was bound to the reprecipitated iron (III) compounds [51].

### 3.4. Effect of Submerged Macrophyte (*Myriophyllum*) on Nutrients Release in Sediments

**Figure 7:**
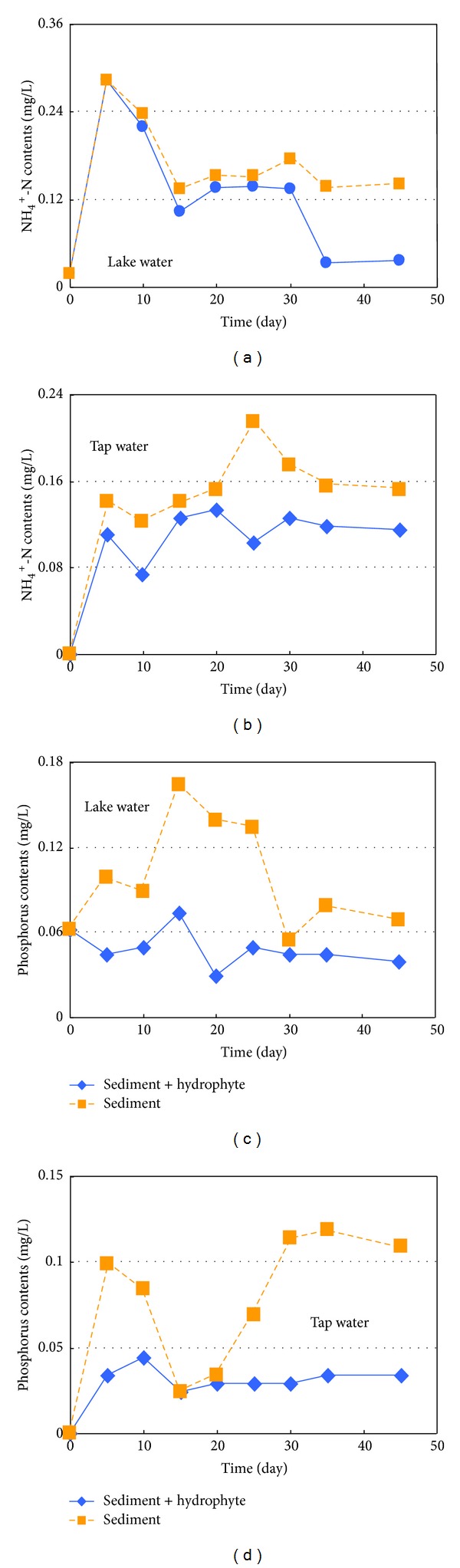
Impacts of hydrophytes (*Myriophyllum*) on nitrogen and phosphorus released from sediments.

The exchanges of nutrients between water and sediment are highly complex, involving interrelated chemical, biological, and physical processes [52, 53]. One of the most important ways in which submerged macrophytes influence lake structure and function is through modification of phosphorus cycling [54]. Submerged macrophytes can reduce the concentration of different phosphorus species in the overlying water, mainly by uptaking the phosphorus from overlying water, inactivating the alkaline phosphatase activity in the sediment and overlying water, reducing sediment resuspension, and controlling the release of internal phosphorus loading [55, 56]. In this study, a common submerged plant species (*Myriophyllum*) was chose to discuss the effects on NH_4_^+^-N and phosphorus release from sediments. As is shown in Figure 7, this submerged aquatic plant can be effectively reduced by the nutrients concentration of lake water. The concentrations of NH_4_^+^-N in lake water declined to a low level after being cultured for 35 days and tended to level off in the later 10 days with the removal rates ranging from 74.1% to 76.5% (Figure 7(a)). Concentrations of NH_4_^+^-N maintained a relatively low level compared to that of only sediment in lake water, which indicated that the abilities of assimilating NH_4_^+^-N of *Myriophyllum* were obvious and effectively inhibited the nitrogen release from sediments. Although plant uptake played a significant role in the removal of NH_4_^+^-N, it did not account for all of NH_4_^+^-N loss from the system, indicating the possibility of biochemical and physicochemical processes functioning in the system. The reductions of NH_4_^+^-N observed in the control samples implied that these processes were at work reducing nitrogen, and this phenomenon is clear in tap water treatment (Figure 7(b)).

The removal effect of *Myriophyllum* on sediment total phosphorus was not significant, mainly due to rooted macrophytes use sediment as their source of phosphorus. In the two experiments and the release of phosphorus from sediment after it was mixed with lake water at the beginning of experiment, total phosphorus of the water increased steadily during the first 15 days, followed by a decrease towards the end of the culture period (Figure 7(c)). The trapping rate of *Myriophyllum *on sediment phosphorus was ranged from 43.4% to 79.2%, the main reason was the abundant phosphorus content of sediment. As a result of the requirements of phosphorus for growth of aquatic plants and the activities of microorganisms were limited, the phosphorus in sediment could not be largely absorbed. *Myriophyllum* had a strong root system and high growth and reproduction ability, which benefited from the absorption of phosphorus from sediment directly. As a whole, the existence of submerged macrophytes reduced the internal loading of total phosphorus in sediments.

### 3.5. Quantitative Assessment for the Trophic State of Lake Daihai

Some major physicochemical variables of the Lake Daihai were listed in Table 4. Basic statistics on Chl-a and other water parameters were summarized, and correlation analysis was constructed (Table 5). The results showed that Chl-a concentration was more or less correlated with other parameters. Just like Chl-a concentration, SD was another important indicator of water quality and was negatively correlated with Chl-a concentration. The other parameters such as total nitrogen and total phosphorus were positively correlated with Chl-a. However, the correlative degree was different between these parameters and Chl-a concentration; total nitrogen and total phosphorus had a high correlation with Chl-a, while water temperature, pH, and COD_Mn_ have a slightly lower correlation with Chl-a.

Since these variables were very commonly included in water monitoring they were treated as key factors for environmental assessment. Aizaki [38] introduced chlorophyll-a as a reference parameter and applied his modified Carlson's trophic state index (TSI_M_) to Japanese lakes of different depths. But weight allocations for evaluating parameters were not determined in this method. Consequently, the comprehensive trophic state index (TSI_C_) based on TSI_M_ was more appropriate for evaluating the trophic states of Chinese shallow lakes [39]. In the present study, fifteen water bodies of Daihai Lake were studied applying these two methods to assess its trophic status. The index for chlorophyll-a, Secchi disk depth, and total phosphorus of TSI_M_ along with the trophic levels of the studied lake are presented in Table 6. From the indictor of TSI_M_ (Chl-a) in the year of 2007, all sampling sites at 30–50 were classified as the mesotrophy state and upper mesotrophic state; obviously, the TSI_M_ (Chl-a) distribution of the studied lake is close to the Chl-a distribution due to their same factors. The TSI_M_ in Daihai Lake has a range of 53.72–70.61 with a mean of 58.43, indicating that most of sampling sites are in eutrophy.

The key factor when identifying trophic type of lake using comprehensive trophic state index (TSI_C_) is that the concentration of Chl-a in water is used as the main reference, and then the different group samples are normalized to obtain the cluster weights of different nutrients parameters. Methods for confirming cluster weight is as follows:

(4) $$\begin{array}{c} x_{ij}^{\prime }=\frac{x_{ij}-m_{i}}{d_{i}}. \end{array}$$

In this equation, *d*_*i*_ = *x*_*i*max⁡_ − *x*_*i*min⁡_, where *x*_*i*max⁡_ and *x*_*i*min⁡_ are the maximum and minimum values of the *i*th parameter; *x*_*ij*_′ is the *j*th pollution index value of the *i*th parameter by normalization. In this study, Chl-a, TP, TN, and SD were chosen as the evaluation parameters; the results of clustering weights of evaluation indexes are shown in the Table 7, and the calculated matrix is as follows: *d* = (0.88  0.74  0.35  0.75  3.62) and *m* = (2.60  0.059  2.52  0.35  17.12). These data are separately substituted into (4), and the can obtain clustering coefficient matrix, which is shown in (5):

(5) $$\begin{array}{c} x=\left|\begin{array}{ccccc} 0.2484 & 0.0935 & 0.3725 & 0.4667 & 0.5155\\ 1.0000 & 0.0090 & 0.7255 & 0.2000 & 0.7938\\ 0.0518 & 0.0180 & 0.7255 & 0.7333 & 0.1031\\ 0.3252 & 0.0090 & 0.4706 & 0.5333 & 0.2474\\ 0.3252 & 0.1151 & 0.3529 & 0.7333 & 0.4433\\ 0.3275 & 0.0000 & 0.1961 & 0.6667 & 0.7526\\ 0.3505 & 0.0234 & 0.5098 & 0.7601 & 1.0000\\ 0.1522 & 0.0090 & 0.8431 & 0.6000 & 0.0825\\ 0.1299 & 3.7E-17 & 0.5098 & 1.0000 & 0.4433\\ 0.4704 & 1.0000 & 0.0000 & 0.0667 & 0.6082\\ 0.0000 & 0.0090 & 0.5294 & 0.0933 & 0.0825\\ 0.7543 & 0.0917 & 1.0000 & 0.0000 & 0.5258\\ 0.2224 & 0.0054 & 0.9412 & 0.3333 & 0.0000 \end{array}\right|. \end{array}$$

The comprehensive trophic state indexes of water quality were presented in Table 8. From single factor, total nitrogen and COD_Mn_ in Daihai Lake water were in the stage of hypereutropher, while total phosphorus and SD were in a status of slightly eutrophication. But as a whole, the TSI_C_(∑) of lake water was ranged from 47.73 to 53.67 with an average of 50.07. The higher values of TSI_C_(∑) at 50–60 can be observed in the southern and northwestern lake, and the lower TSI_C_(∑) can be noted in the middle of Lake Daihai. Therefore, the accumulation of nutrients in these parts of Daihai Lake should be concerned sufficiently. 

Eutrophication, although a natural process overtime, is often accelerated by human activities termed cultural eutrophication. Humans influence a lake by increasing contents of plant nutrients, primary nitrogen, and phosphorus. These nutrients can enter the lake through agricultural land, sewage, or waste water which can cause overenrichment. This produces direct and indirect biological changes to lakes leading to the production of algal blooms. Consequently, monitoring eutrophication is an important part of assessing and managing lake ecosystems. These two methods mentioned in this study can be used to identify certain conditions in the lake which are related to the factor that limit algal biomass or affect the water quality parameters.

## 4. Conclusions

Nitrogen and phosphorus concentrations in the sediments of the Lake Daihai are influenced by the following factors: hydrochemical and hydrodynamic conditions in water column above the sediment, depth, oxygenation of near bottom water, and physicochemical properties of the sediments. Total phosphorus concentrations in surface sediments was highest in site of DH-14 and decreased gradually from the north to the south of the lake. Concentrations of total nitrogen, NH_4_^+^-N, and NO_3_^−^-N in surface sediment were in the range of 0.27–1.78 g/kg, 2.56–14.5 mg/kg, and 1.79–12.5 mg/kg, respectively. The NH_4_^+^-N content in surface sediments remained higher, and its relative contribution to inorganic nitrogen is over 50%. Relatively low ratio of N/P (0.42–2.59) in water column suggested that the limiting nutrient for the algal growth in the Lake Daihai is N.

Laboratory studies were conducted to determine the effects of environment factors on the release of nitrogen and phosphorus in lake sediment, and the study revealed that the increased release rates of nitrogen and phosphorus would be presented under reductive conditions and high pH values. Moreover, light conditions also indirectly affected nutrients release processes; light exposure could facilitate algae growth and therefore reduced nitrogen and phosphorus concentrations in water column and indirectly restrained the release of nutrients from sediment. Hydrophytes (e.g., *Myriophyllum*) could control these nutrients release from the surface sediments, purify the water body, and prevent the lake eutrophication. Hydrophytes had a strong root system and high growth and reproduction ability, which benefited from the absorption of phosphorus and nitrogen from sediment directly.

Determining trophic state index is an important aspect of lakes survey and is an aspect of water quality. In the present study, modified Carlson's trophic state index (TSI_M_) and comprehensive trophic state index (TSI_C_) were applied to ascertain the trophic classification of the studied lake, and the results showed that the values of TSI_M_ and TSI_C_ ranged from 53.72 to 70.61 and from 47.73 to 53.67, respectively. It is evident from the finding of the present study that the Lake Daihai is facing more stress of cultural eutrophication or manual cleaning of macrophytes and that algal biomass is needed to protect water body from further degradation.

## Figures and Tables

**Table 1:** The expressions of parameters of the trophic state index.

Series numbers	Calculation formulas
1	TLI(chl-a) = 10[2.5 + 1.086 ln(chl-a)] = 10[2.5 + 0.995 ln(chl-a)/ln2.5]
2	TLI(TP) = 10[9.436 + 1.624 ln(TP)] = 10[9.436 + 1.488 ln(TP)/ln2.5]
3	TLI(TN) = 10[5.453 + 1.694 ln(TN)] = 10[5.453 + 4.552 ln(TN)/ln2.5]
4	TLI(SD) = 10[5.118 − 1.941 ln(SD)] = 10[5.118 − 1.778 ln(SD)/ln2.5]
5	TLI(COD) = 10[0.109 + 2.661 ln(COD)] = 10[0.109 + 2.438 ln(COD)/ln2.5]
6	TLI(BOD5) = 10[2.118 + 2.579 ln(BOD)] = 10[2.118 + 2.363 ln(BOD)/ln2.5]
7	TLI(NH_4_^+^-N) = 10[7.77 + 1.649 ln(NH_4_^+^-N)] = 10[7.77 + 1.511 ln(NH_4_^+^-N)/ln2.5]

**Table 2:** Physical and chemical characteristics of the water and sediments.

Items	Range	Mean	SD
Water Characteristics			
pH	8.18–8.84	8.45	0.38
Temperature (°C) (September)	21.5–24.1	23.1	0.84
Dissolved oxygen (mg/L)	2.81–4.83	4.01	0.58
Chl-a (mg/L)	2.60–3.48	2.91	0.72
Total N (mg/L)	2.52–2.87	2.72	0.11
Total P (mg/L)	0.06–0.80	0.13	0.19
Salinity (g/kg)	0.23–0.44	0.33	0.16
Secchi (m)	0.35–1.10	0.81	0.32
COD_Mn_ (mg/L)	17.12–20.73	18.61	1.12
Electric conductivity (ms/cm)	8.19–9.78	9.14	0.53
Sediment characteristics			
Water content (%)	45.4–71.3	63.6	11.7
Clay (%)	3.72–9.53	6.58	1.39
Silt (%)	62.6–86.1	76.1	9.38
Sand (%)	8.04–36.19	25.3	7.49
Organic carbon (g/kg)	6.84–23.5	14.9	8.61

**Table 3:** The pearson correlation coefficients between C, N, P, and different ratios of nutrients (*n* = 15).

	TOC	OM	TN	NH_4_^+^-N	NO_3_^−^-N	TP	IN	C : N	N : P
TOC	1.000							0.373	0.785**
OM	0.999**	1.000						0.327	0.751**
TN	0.939**	0.931**	1.000					0.037	0.872**
NH_4_^+^-N	0.293	0.291	0.248	1.000				0.194	0.294
NO_3_^−^-N	−0.195	−0.189	0.021	−0.149	1.000			−0.748*	0.069
TP	0.675*	0.669*	0.633*	0.065	−0.134	1.000		0.250	0.176
IN	0.153	0.148	0.238	0.822**	0.443	−0.018	1.000	−0.254	0.306

TOC: total organic carbon; OM: organic matter; TN: total nitrogen; TP: total phosphorus; NH_4_^+^-N: ammonia nitrogen.

NO_3_^−^-N: nitrate nitrogen; TP: total phosphorus; IN: inorganic nitrogen.

*Correlation is significant at the 0.05 level (2 tailed).

**Correlation is significant at the 0.01 level (2 tailed).

**Table 4:** Major physicochemical parameters of water quality in the Daihai Lake.

Sampling sites	Chl-a (mg/m^3^)	COD_Mn_ (mg/L)	NH_4_^+^-N (mg/L)	NO_2_-N (mg/L)	NO_3_-N (mg/L)	TN (mg/L)	TP (mg/L)	SD (m)	pH	WT (°C)
DH-1	2.82	18.98	0.0254	0.0033	0.171	2.65	0.1286	0.71	8.81	23.1
DH-2	3.48	19.99	0.0197	0.0029	0.131	2.78	0.0661	0.51	8.47	23.6
DH-3	3.18	16.29	0.0217	0.0042	0.101	2.43	0.0673	0.69	8.33	23.3
DH-4	2.65	17.49	0.0292	0.0003	0.115	2.78	0.0727	0.89	8.18	23.5
DH-5	2.89	18.01	0.0121	0.0005	0.109	2.69	0.0659	0.75	8.34	24.1
DH-6	2.89	18.72	0.0114	0.0007	0.123	2.65	0.1446	0.92	8.82	24.3
DH-7	2.89	19.84	0.0075	0.0017	0.117	2.59	0.0594	0.85	8.45	23.4
DH-8	2.91	20.73	0.0248	0.0021	0.121	2.71	0.0767	0.91	8.07	22.8
DH-9	2.73	17.41	0.0122	0.0019	0.113	2.82	0.0661	0.81	8.84	22.5
DH-10	2.71	18.72	0.0690	0.0029	0.118	2.69	0.0594	1.09	8.87	23.6
DH-11	3.01	19.32	0.0158	0.0023	0.116	2.52	0.7996	0.42	8.15	22.3
DH-12	2.61	17.79	0.0095	0.0051	0.112	2.71	0.0671	0.85	8.48	22.7
DH-13	2.77	17.41	0.0478	0.0345	0.119	2.71	0.0642	0.45	8.37	22.5
DH-14	3.26	19.02	0.0189	0.0027	0.116	2.87	0.1273	0.35	8.32	21.7
DH-15	2.79	17.12	0.0177	0.0091	0.116	2.85	0.0631	0.64	8.01	21.8

Average	2.89	18.61	0.0185	0.0049	0.121	2.72	0.1332	0.72	8.44	22.9

SD: Secchi disk depth; WT: water temperature; TN: total nitrogen; TP: total phosphorus.

**Table 5:** Summary statistics on Chl-a and the other water parameters measured in Daihai Lake (*n* = 15).

Parameters	Chl-a	COD_Mn_	NH_4_^+^-N	NO_2_-N	NO_3_-N	TN	TP	SD	pH	WT
Chl-a	1.000									
COD_Mn_	0.338	1.000								
NH_4_^+^-N	−0.212	−0.063	1.000							
NO_2_-N	−0.160	−0.312	0.413	1.000						
NO_3_-N	0.018	0.395	0.098	−0.015	1.000					
TN	0.602*	0.029	0.038	0.071	0.072	1.000				
TP	0.646*	0.226	−0.149	−0.117	0.009	−0.396	1.000			
SD	−0.587*	0.017	0.189	−0.395	−0.046	−0.09	−0.41	1.000		
pH	−0.171	0.009	0.204	−0.131	0.382	−0.041	−0.225	0.397	1.000	
WT	−0.009	0.110	0.051	−0.326	0.071	−0.361	−0.245	0.570*	0.424	1.000

COD: chemical oxygen demand; TN: total nitrogen; TP: total phosphorus; SD: secchi disk depth; WT: water temperature.

*Correlation is significant at the 0.05 level (2 tailed).

**Table 6:** Appraisement results of TSI_M_ on water quality of the Lake Daiahi.

Sampling sites	TSI_M_ (SD)	TSI_M_ (Chl-a)	TSI_M_ (TP)	*∑*TSI_M_
DH-1	70.83	35.91	72.09	59.61
DH-2	76.45	38.21	63.72	59.46
DH-3	69.82	37.19	65.21	56.81
DH-4	66.63	35.22	64.92	55.59
DH-5	69.67	36.17	63.71	56.52
DH-6	66.63	36.23	73.56	58.78
DH-7	67.58	36.21	62.38	55.38
DH-8	66.26	36.29	65.62	56.04
DH-9	68.61	35.58	63.71	55.96
DH-10	63.28	35.51	62.38	53.72
DH-11	80.17	36.63	95.02	70.61
DH-12	67.58	35.03	63.72	55.44
DH-13	79.36	35.73	63.46	59.51
DH-14	82.40	37.51	71.96	63.69
DH-15	73.40	35.82	63.28	57.47

Average	71.35	36.14	67.82	58.43

**Table 7:** Cluster weights of different water parameters from the Lake Daihai.

Parameters	Chl-a	TP	TN	SD	COD_Mn_
*R*_*ij*_	1.0000	0.1887	0.1249	−0.5917	0.5908
*R*_*ij*_^2^	1.0000	0.0356	0.0156	0.3501	0.3491
*W*_*j*_	0.5713	0.0203	0.0089	0.2001	0.1994

**Table 8:** Comprehensive trophic status index (TSI_C_) of lake eutrophication at different sampling sites of Lake Daihai.

Sampling sites	TSI_C_ (Chl-a)	TSI_C_ (TP)	TSI_C_ (TN)	TSI_C_ (SD)	TSI_C_ (COD_Mn_)	TSI_C_ (*∑*)
DH-1	36.25	61.05	71.07	58.12	79.41	50.05
DH-2	38.54	50.22	71.83	64.63	80.79	52.72
DH-3	36.48	55.52	71.45	58.71	78.85	50.07
DH-4	35.57	51.78	71.83	53.23	77.24	48.06
DH-5	36.51	50.22	71.28	56.76	78.02	49.43
DH-6	36.54	62.95	71.02	53.23	79.05	49.18
DH-7	36.52	48.49	70.67	54.33	80.59	49.42
DH-8	36.61	52.65	71.37	52.82	81.77	49.48
DH-9	35.92	50.22	70.08	55.51	77.12	48.67
DH-10	35.85	48.49	71.37	49.33	79.06	47.73
DH-11	36.98	90.73	71.21	68.97	79.88	53.32
DH-12	35.38	50.22	71.39	54.33	77.69	48.23
DH-13	36.07	49.89	71.41	68.02	77.12	51.24
DH-14	37.85	60.88	71.44	71.56	79.47	53.67
DH-15	36.17	49.55	72.29	61.11	76.66	49.82
